# Supplementary material for: Strategy to mitigate substrate inhibition in wastewater treatment systems
Source: Nat Commun. 2024 Sep 10;15:7920. doi: 10.1038/s41467-024-52364-9 (PMC11387818; doi:10.1038/s41467-024-52364-9)
Supplement: Supplementary file 1 — Supplementary Information [file 41467_2024_52364_MOESM1_ESM.pdf]

Supplementary Information

Strategy to Mitigate Substrate Inhibition in Wastewater Treatment Systems

Author list

Beiying Li[a], Conghe Liu[a], Jingjing Bai[a], Yikun Huang[a], Run Su[a], Yan Wei[b], Bin Ma[a,*]

Affiliations

a Key Laboratory of Agro-Forestry Environmental Processes and Ecological Regulation of Hainan Province, School of Environmental Science and Engineering, Hainan University, Haikou 570228, China

b State Key Laboratory of Marine Resources Utilization in the South China Sea, Hainan University, Haikou, 570228, China

The following are included as supporting information for this paper:

Number of pages: 10

Number of tables: 1

Number of figures: 5

[*] Corresponding author.

E-mail addresses: mabin@hainanu.edu.cn (B. Ma).

Supplementary Methods: Nitrite treatment concentrations in the side-stream unit

One-tenth of the sludge was exposed to nitrite in the sidestream unit each time, and ten days for each operation of the unit. To avoid the complete loss of microbial activity in the side-stream unit, the operation time of the side-stream unit was set to 8 h. After nitrite exposure, the sludge was returned to mix with the remaining sludge in UASB2 reactor, and operated at the normal influent concentration for 16 h. The nitrogen removal rate (NRR) of the UASB2 reactor was measured after 16 h operation. Compared to the previous day, the nitrite concentration on the subsequent day increased by10 mg·L^{-1} if the NRR of the reactor did not decrease after nitrite exposure. A total of three operations of the side-stream treatment unit.

Supplementary Result

Compared with the UASB1 sludge, the proportion of *Chloroflexi* and *Bacteroidotes* in UASB2 increased to 16.77% and 19.31%, respectively. *Chloroflexi* and *Bacteroidota* have the ability to degrade complex polymers [1], *Chloroflexi* acts as a skeleton for the aggregation of microorganism and *Bacteroidota* employ several unique strategies to further enhance the efficiency of their carbohydrate metabolism [2,3]. It can be used to explain why the system maintains a high sludge activity with an enough long sludge age in this study.

Analyses using Venn diagrams yielded 20 genera unique to UASB2 (Fig. S2). Among them, *Sphaerotilus* was Gram-negative organisms which involved in biofilm formation [4]. *Norank_Saprospiraceaeare* was the main EPS degradation bacteria, the degradation of EPS might also be the carbon source for the denitrification [5, 6, 7]. *Caulobacteraceae_norank* coordinates the cell division cycle and multiple cellular differentiation, enabling organisms to cope with a wide range of environmental fluctuations [8].

Supplementary Table 1. Variation of nitrite concentration in the sidestream unit.

Phase	Days	nitrite concentration in the sidestream unit of UASB2 ($mg\cdot L^{-1}$)	nitrite concentration in the sidestream unit of UASB3 ($mg\cdot L^{-1}$)
II	1	30	30
	2	35	40
	3	30	30
	4	35	30
	5	30	30
	6	30	40
	7	30	30
	8	30	30
	9	30	30
	10	30	30
IV	1	30	30
	2	30	30
	3	30	30
	4	30	30
	5	35	40
	6	35	50
	7	35	60
	8	40	60
	9	40	70
	10	45	70
IV	1	30	30
	2	30	30
	3	35	40
	4	40	50
	5	45	60
	6	50	70
	7	55	80
	8	60	90
	9	65	100
	10	60	100

Supplementary Figures

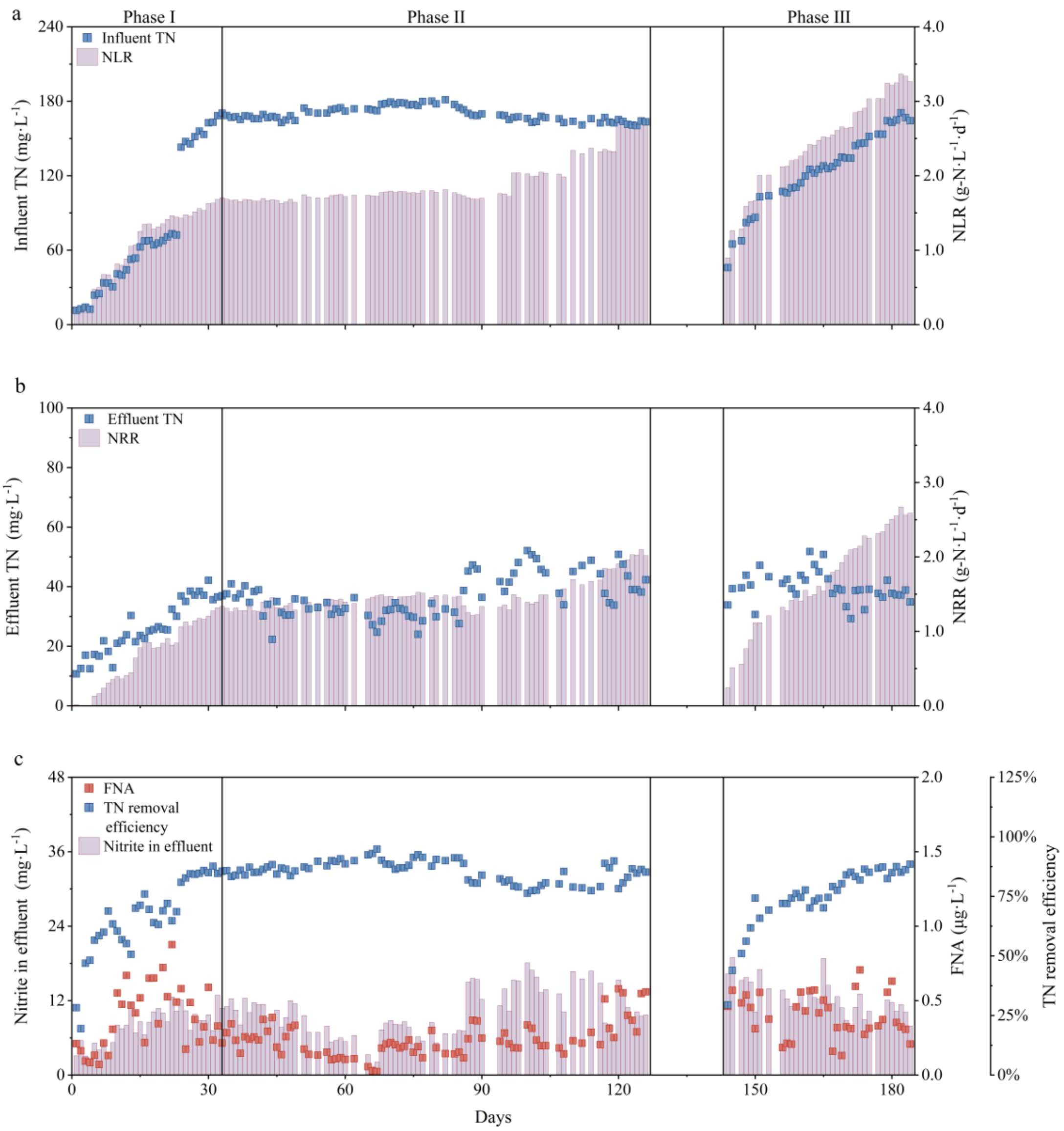

Supplementary Fig. 1. Reactor performances of the UASB1 reactor: (a) influent TN concentrations and NLR; (b) effluent TN concentrations and NRR; (c) effluent nitrite concentrations, FNA and TN removal efficiency.

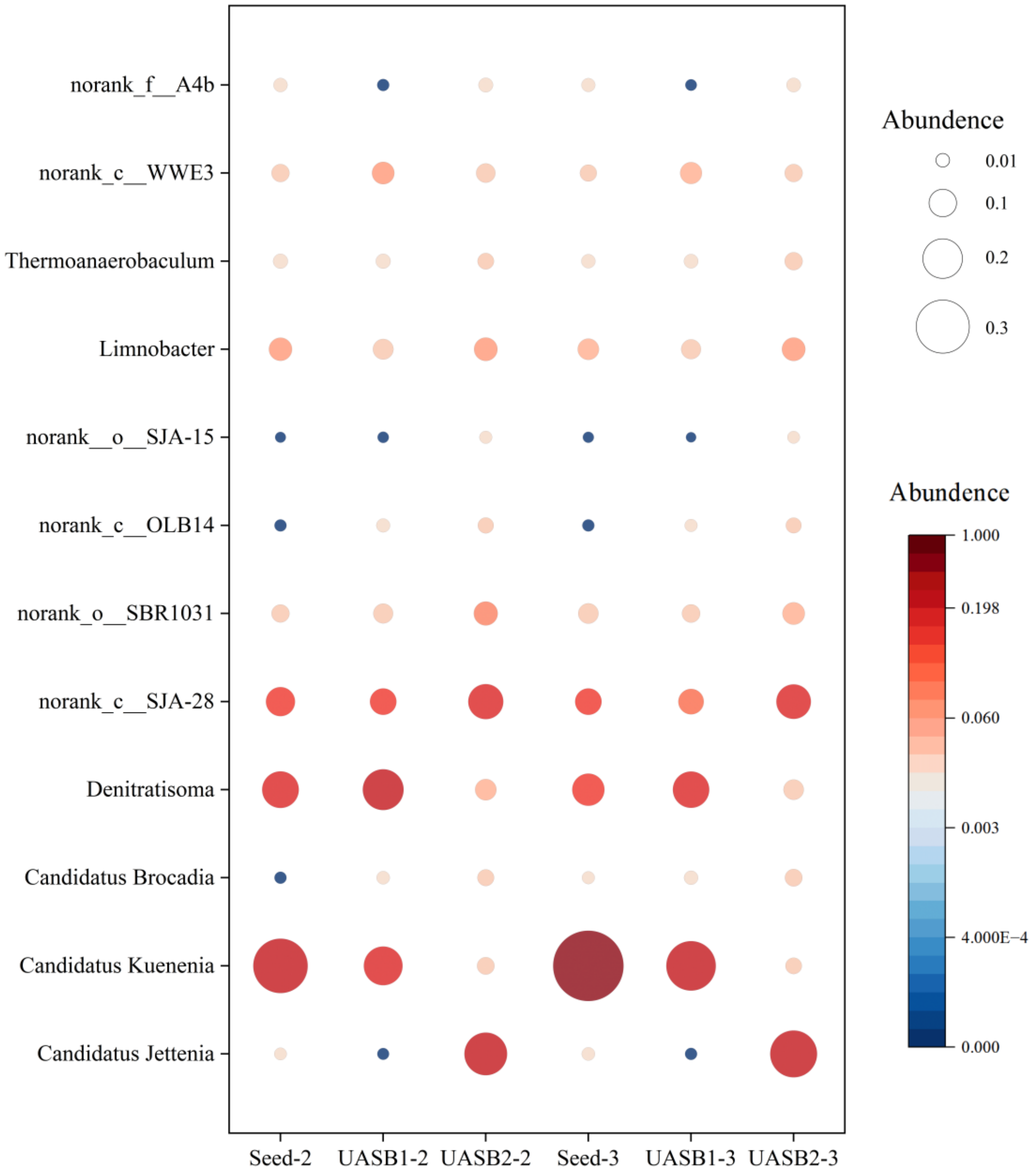

Supplementary Fig. 2. Analysis of bacterial community abundance at the genus level from the 16S amplicon sequencing. Seed-2, UASB-2, and UASB-3 were from the second sequencing, and Seed-3, UASB-3, and UASB-3 were from the third sequencing. Each sample was sequenced by three times, and the first sequencing data was shown in Fig.6.

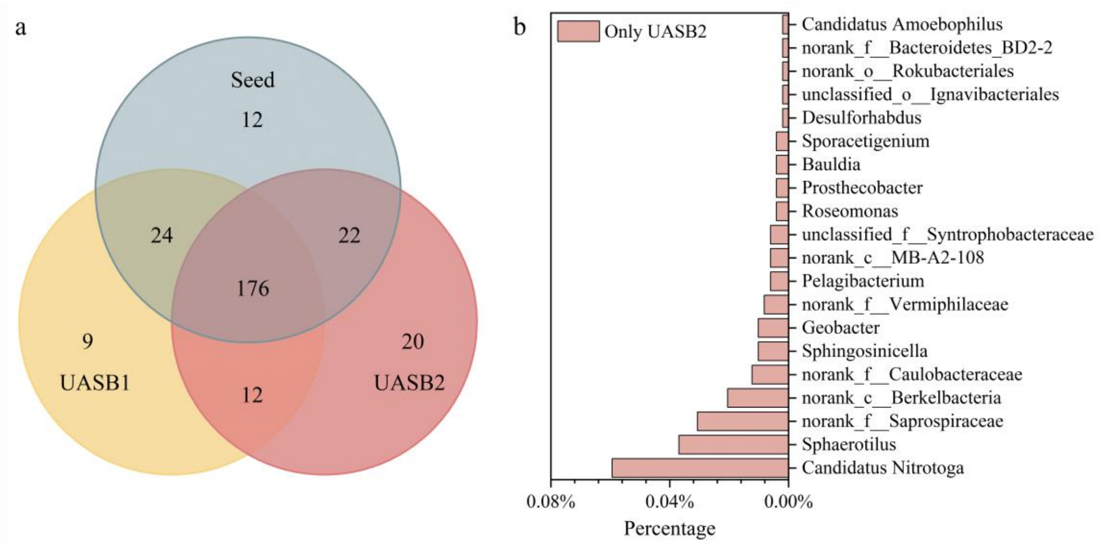

Supplementary Fig. 3. (a) The number of unique and shared genera identified across the three different sample; (b) the abundance of Unique genera in UABS2.

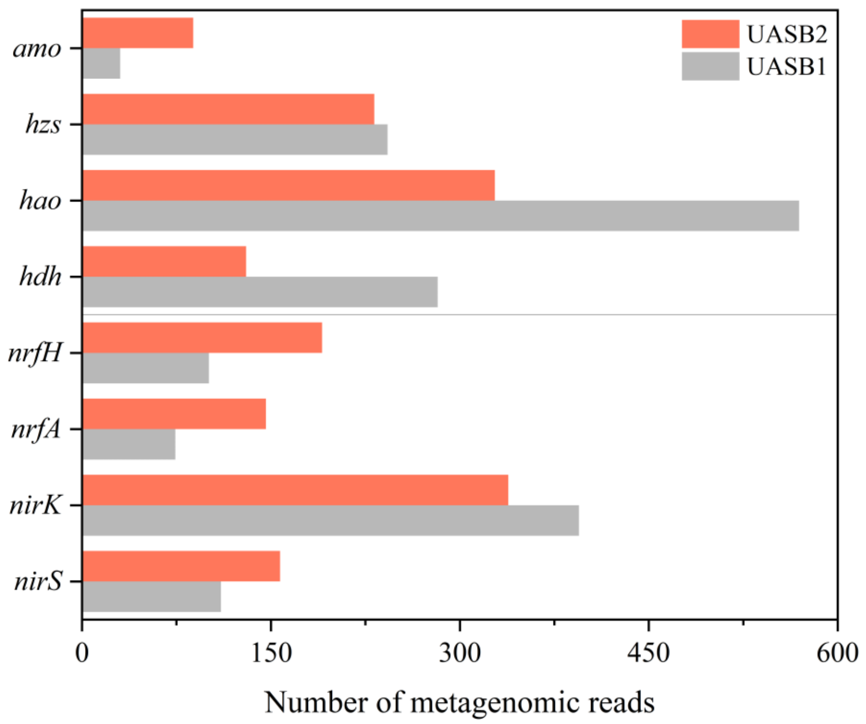

Supplementary Fig. 4. Number of genes reads associated with the anammox process from metagenomic sequencing.

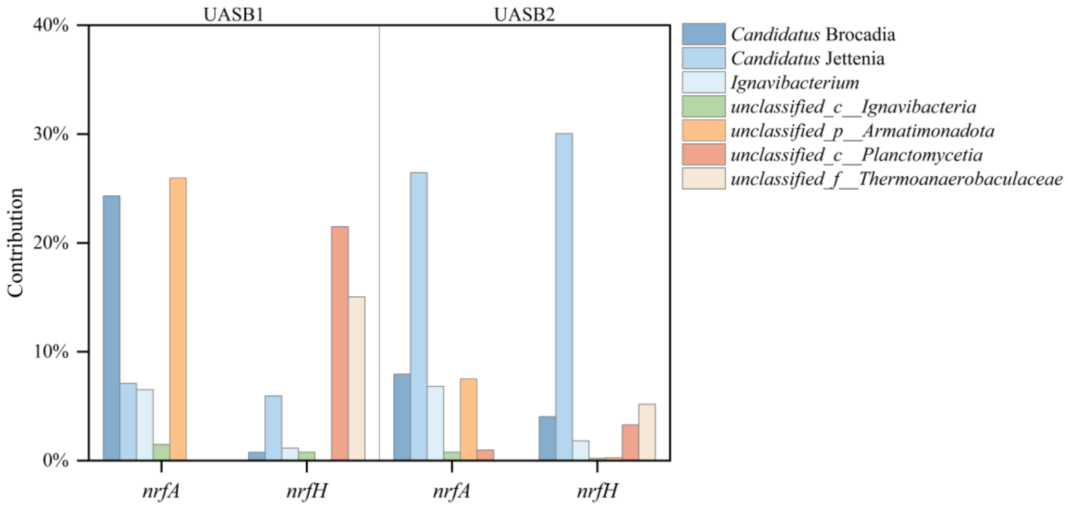

Supplementary Fig. 5. The contribution of bacterial community to *nrf*AH gene, the left side was untreated with nitrite, the right side was treated with nitrite.

Supplementary Reference

1. Lu C-Y, Yuan C, Zhu T, Wang Y. Effect of humic acid on the single-stage nitrogen removal using anammox and partial nitritation (SNAP) process: performance and bacterial communities. *Journal of Environmental Chemical Engineering* **9**, 106680 (2021).

2. Fernández-Gómez B, *et al.* Ecology of marine Bacteroidetes: a comparative genomics approach. *The ISME Journal* **7**, 1026-1037 (2013).

3. Gavriilidou A, *et al.* Comparative genomic analysis of Flavobacteriaceae: insights into carbohydrate metabolism, gliding motility and secondary metabolite biosynthesis. *BMC Genomics* **21**, 569 (2020).

4. Mähler B, *et al.* Adipocere formation in biofilms as a first step in soft tissue preservation. *Scientific Reports* **12**, 10122 (2022).

5. Zhang M, Yao J, Wang X, Hong Y, Chen Y. The microbial community in filamentous bulking sludge with the ultra-low sludge loading and long sludge retention time in oxidation ditch. *Scientific Reports* **9**, 13693 (2019).

6. He J, Xu J, Yu H. Performance and bacterial community dynamics of aerobic granular sludge working at low temperature enhanced by melamine framework embedding. *Journal of Environmental Chemical Engineering* **9**, 105156 (2021).

7. Guo Y, Peng Y, Wang B, Li B, Zhao M. Achieving simultaneous nitrogen removal of low C/N wastewater and external sludge reutilization in a sequencing batch reactor. *Chemical Engineering Journal* **306**, 925-932 (2016).

8. Panis G, Lambert C, Viollier PH. Complete genome sequence of Caulobacter crescentus bacteriophage φCbK. *Journal of virology* **86**, 10234-10235 (2012).
