# Supplementary material for: Strategy to mitigate substrate inhibition in wastewater treatment systems
Source: Nat Commun. 2024 Sep 10;15:7920. doi: 10.1038/s41467-024-52364-9 (PMC11387818; doi:10.1038/s41467-024-52364-9)
Supplement: Supplementary file 3 — Reporting Summary [file 41467_2024_52364_MOESM3_ESM.pdf]

nature portfolio

Corresponding author(s): Bin Ma

Last updated by author(s): Aug 29, 2024

Reporting Summary

Nature Portfolio wishes to improve the reproducibility of the work that we publish. This form provides structure for consistency and transparency in reporting. For further information on Nature Portfolio policies, see our Editorial Policies and the Editorial Policy Checklist.

Statistics

For all statistical analyses, confirm that the following items are present in the figure legend, table legend, main text, or Methods section.

n/a	Confirmed	
☐	☒	The exact sample size (*n*) for each experimental group/condition, given as a discrete number and unit of measurement
☐	☒	A statement on whether measurements were taken from distinct samples or whether the same sample was measured repeatedly
☒	☐	The statistical test(s) used AND whether they are one- or two-sided<br>*Only common tests should be described solely by name; describe more complex techniques in the Methods section.*
☒	☐	A description of all covariates tested
☒	☐	A description of any assumptions or corrections, such as tests of normality and adjustment for multiple comparisons
☒	☐	A full description of the statistical parameters including central tendency (e.g. means) or other basic estimates (e.g. regression coefficient) AND variation (e.g. standard deviation) or associated estimates of uncertainty (e.g. confidence intervals)
☒	☐	For null hypothesis testing, the test statistic (e.g. *F*, *t*, *r*) with confidence intervals, effect sizes, degrees of freedom and *P* value noted<br>*Give P values as exact values whenever suitable.*
☒	☐	For Bayesian analysis, information on the choice of priors and Markov chain Monte Carlo settings
☒	☐	For hierarchical and complex designs, identification of the appropriate level for tests and full reporting of outcomes
☒	☐	Estimates of effect sizes (e.g. Cohen's *d*, Pearson's *r*), indicating how they were calculated

Our web collection on statistics for biologists contains articles on many of the points above.

Software and code

Policy information about availability of computer code

Data collection	No software was used.
Data analysis	Microbiological analiyses based on the Majorbio Cloud Platform (www.majorbio.com), such as Mothur (v.1.30.2).

For manuscripts utilizing custom algorithms or software that are central to the research but not yet described in published literature, software must be made available to editors and reviewers. We strongly encourage code deposition in a community repository (e.g. GitHub). See the Nature Portfolio guidelines for submitting code & software for further information.

Data

Policy information about availability of data

All manuscripts must include a data availability statement. This statement should provide the following information, where applicable:

- Accession codes, unique identifiers, or web links for publicly available datasets
- A description of any restrictions on data availability
- For clinical datasets or third party data, please ensure that the statement adheres to our policy

All data generated in this study are provided in the article file, Supplementary Information, and Supplementary Data. The relevant source date from each figure are provided in the Source Data files. The amplicon sequence datassets generated during this study are available with accession number PRJNA1102518. The metagenomic sequence data could be found at BioProject PRJNA1094462. Source data are provided with this paper.

Research involving human participants, their data, or biological material

Policy information about studies with human participants or human data. See also policy information about sex, gender (identity/presentation), and sexual orientation and race, ethnicity and racism.

Reporting on sex and gender	No involved
Reporting on race, ethnicity, or other socially relevant groupings	No involved
Population characteristics	No involved
Recruitment	No involved
Ethics oversight	No involved

Note that full information on the approval of the study protocol must also be provided in the manuscript.

Field-specific reporting

Please select the one below that is the best fit for your research. If you are not sure, read the appropriate sections before making your selection.

☐ Life sciences ☐ Behavioural & social sciences ☒ Ecological, evolutionary & environmental sciences

For a reference copy of the document with all sections, see nature.com/documents/nr-reporting-summary-flat.pdf

Ecological, evolutionary & environmental sciences study design

All studies must disclose on these points even when the disclosure is negative.

Study description	our study aims to investigate a feasible approach to enhance antifragility of wastewater treatment systems by creating a non-lethal high substrate environment. We employed a controlled trial to evaluate the tolerance of bacterial community and resistance of system.
Research sample	anammox sludge, which was enriched in the laboratory.
Sampling strategy	water samples were taken according to experimental objectives, microbiological samples were taken at beginning and end of the experiment, respectively.
Data collection	After each sample was taken, it was analysed and recorded the date by Beiying Li and Conghe Liu.
Timing and spatial scale	The experimental period was 184 days, and water samples were taken daily while the reactor was in operation.
Data exclusions	No data were excluded from the analses.
Reproducibility	The reactor was not operation repeatedly, sample was tested repeated.
Randomization	Anammox sludge was allocated using randomization process.
Blinding	Microbial research is not applicable to blinding.

Did the study involve field work? ☐ Yes ☒ No

Reporting for specific materials, systems and methods

We require information from authors about some types of materials, experimental systems and methods used in many studies. Here, indicate whether each material, system or method listed is relevant to your study. If you are not sure if a list item applies to your research, read the appropriate section before selecting a response.

Materials & experimental systems

n/a	Involved in the study
☒	☐ Antibodies
☒	☐ Eukaryotic cell lines
☒	☐ Palaeontology and archaeology
☒	☐ Animals and other organisms
☒	☐ Clinical data
☒	☐ Dual use research of concern
☒	☐ Plants

Methods

n/a	Involved in the study
☒	☐ ChIP-seq
☒	☐ Flow cytometry
☒	☐ MRI-based neuroimaging

Plants

Seed stocks	we do not use plants in our study.
Novel plant genotypes	we do not use these methods.
Authentication	we do not use these procedures.
